# Protein Kinase C-δ Mediates Shedding of Angiotensin-Converting Enzyme 2 from Proximal Tubular Cells

**DOI:** 10.3389/fphar.2016.00146

**Published:** 2016-06-01

**Authors:** Fengxia Xiao, Joseph Zimpelmann, Dylan Burger, Christopher Kennedy, Richard L. Hébert, Kevin D. Burns

**Affiliations:** Division of Nephrology, Department of Medicine, Kidney Research Centre, Ottawa Hospital Research Institute, University of OttawaOttawa, ON, Canada

**Keywords:** ACE2, kidney, diabetes, ADAM17, PKC, sheddase, renin-angiotensin system

## Abstract

Angiotensin-converting enzyme 2 (ACE2) degrades angiotensin (Ang) II to Ang-(1–7), and protects against diabetic renal injury. Soluble ACE2 fragments are shed from the proximal tubule, and appear at high levels in the urine with diabetes. High glucose-induced shedding of ACE2 from proximal tubular cells is mediated by the enzyme “a disintegrin and metalloproteinase-17″ (ADAM17). Here, we investigated the mechanism for constitutive shedding of ACE2. Mouse proximal tubular cells were cultured and ACE2 shedding into the media was assessed by enzyme activity assay and immunoblot analysis. Cells were incubated with pharmacologic inhibitors, or transfected with silencing (si) RNA. Incubation of proximal tubular cells with increasing concentrations of D-glucose stimulated ACE2 shedding, which peaked at 16 mM, while L-glucose (osmotic control) had no effect on shedding. In cells maintained in 7.8 mM D-glucose, ACE2 shedding was significantly inhibited by the pan-protein kinase C (PKC) competitive inhibitor sotrastaurin, but not by an inhibitor of ADAM17. Incubation of cells with the PKC-α and -β1-specific inhibitor Go6976, the PKC β1 and β2-specific inhibitor ruboxistaurin, inhibitors of matrix metalloproteinases-2,-8, and -9, or an inhibitor of ADAM10 (GI250423X) had no effect on basal ACE2 shedding. By contrast, the PKC-δ inhibitor rottlerin significantly inhibited both constitutive and high glucose-induced ACE2 shedding. Transfection of cells with siRNA directed against PKC-δ reduced ACE2 shedding by 20%, while knockdown of PKC-ε was without effect. These results indicate that constitutive shedding of ACE2 from proximal tubular cells is mediated by PKC-δ, which is also linked to high glucose-induced shedding. Targeting PKC-δ may preserve membrane-bound ACE2 in proximal tubule in disease states and diminish Ang II-stimulated adverse signaling.

## Introduction

The renal proximal tubular renin-angiotensin system (RAS) is activated in pathogenic states such as diabetic nephropathy, and enhanced generation of the vasoconstrictor peptide angiotensin (Ang) II contributes to progressive nephron injury. Thus, Ang II causes cell hypertrophy, reactive oxygen species generation, inflammation, and apoptosis in proximal tubule, which promote development of chronic tubulointerstitial fibrosis (Sachse and Wolf, 2007). To counteract RAS activation, the proximal tubule expresses angiotensin-converting enzyme 2 (ACE2), a type I membrane protein localized primarily to the apical membrane, where it cleaves the carboxy-terminal amino acid of Ang II to form the heptapeptide Ang-(1–7) (Warner et al., 2005; Shaltout et al., 2007). Not only does ACE2 reduce local levels of Ang II, but the generation of Ang-(1–7) may provide renoprotection via interaction with its Mas receptor (Santos et al., 2003), inducing downstream inhibition of Ang II signaling responses (Su et al., 2006). Thus, gene deletion of ACE2 or pharmacologic ACE2 inhibition worsens albuminuria and promotes loss of renal function in diabetic mouse models (Soler et al., 2007; Wong et al., 2007), while overexpression of ACE2 or exogenous delivery of human recombinant ACE2 to diabetic mice reduces albuminuria and kidney injury (Oudit et al., 2010; Nadarajah et al., 2012). Furthermore, studies in kidney biopsy specimens from humans with diabetic nephropathy reveal diminished expression of ACE2 in glomeruli and tubular components, suggesting that loss of ACE2 may contribute to disease progression (Reich et al., 2008).

Soluble active fragments of ACE2 appear in the urine in subjects with diabetes and chronic kidney disease (Mizuiri et al., 2011; Xiao et al., 2012; Park et al., 2013; Cherney et al., 2014), and are thought to be shed from proteolytic cleavage of the ACE2 ectodomain. These studies implicate urinary ACE2 as a potential biomarker of early diabetic nephropathy. In this regard, we have demonstrated that ACE2 cleavage in mouse proximal tubular cells in conditions of high glucose involves activation of a disintegrin and metalloproteinase 17 (ADAM17), a zinc-dependent protease also known as tumor necrosis factor-α-converting enzyme (TACE) (Xiao et al., 2014). ADAM17 not only cleaves ACE2, but is also involved as a sheddase enzyme for other target proteins, including tumor necrosis factor-α and epidermal growth factor (EGF) receptor ligands (Hinkle et al., 2004). ACE2 cleavage by ADAM17 has also been demonstrated in cultured HEK293, HK-2 proximal tubular, Huh7, COS7, and human respiratory epithelial cells (Lambert et al., 2005; Jia et al., 2009; Salem et al., 2014; Grobe et al., 2015). In diabetic Akita mice, insulin treatment decreases renal ADAM17 protein expression, associated with reduced urinary ACE2 shedding (Salem et al., 2014). In diabetic states therefore, ADAM17 activation may be responsible for increased urinary shedding of ACE2.

In addition to the ADAM family of metalloproteases, ectodomain shedding of proteins may be regulated by several stimuli, including protein kinase C (PKC), calcium ionophores, G-protein-coupled receptors, and mitogen-activated protein kinases (Murphy, 2009). Indeed, ADAM17 is considered to be the major sheddase responsive to PKC stimulation by phorbol esters (Kveiborg et al., 2011). However, in our previous studies we showed that while ADAM17 is involved in the shedding of ACE2 fragments from proximal tubular cells in conditions of high glucose, constitutive (basal) shedding of ACE2 is not affected by inhibition of ADAM17 (Xiao et al., 2014). Therefore the purpose of the present studies was to explore the mechanisms responsible for constitutive shedding of ACE2 from proximal tubule. Our results reveal that the PKC-δ isoform is a master regulator of both constitutive and high glucose-stimulated shedding of ACE2 fragments from cultured mouse proximal tubular cells.

## Materials and Methods

### Primary Cultures of Mouse Proximal Tubular Cells

The study involved preparations of primary cultures of mouse proximal tubular cells derived from male C57BL6 mice (Charles River Laboratories, Saint-Constant, QC, Canada). All mice were housed according to the Canadian Council on Animal Care (CCAC) guidelines. The experimental protocols were approved by the Animal Care Committee at the University of Ottawa (study protocol ME-2405).

For each experiment, primary cultures of mouse proximal tubular cells were prepared from 2 male C57BL6 mice (four kidneys, age of mice 12–16 weeks) by collagenase digestion of renal cortices followed by Percoll gradient centrifugation, as we have described (Xiao et al., 2014). After centrifugation in Percoll, the fourth band (F4), enriched in proximal tubular segments, was aspirated, suspended in culture medium and centrifuged to remove the Percoll solution. The final proximal tubular pellet was suspended in culture medium and seeded onto 35-mm culture dishes.

Cells were initially grown in a defined medium of DMEM-F12 (1:1), supplemented with insulin (5 μg/ml), transferrin (5 μg/ml), selenium (5 ng/ml), hydrocortisone (50 nM), 3,3′,5-triiodo-*L*-thyronine (2.5 nM), 100 U/ml penicillin, 100 mg/ml streptomycin, and 10% fetal bovine serum (FBS). After 24 h, cells were switched to a defined serum-free medium, with D-glucose concentration for most experiments set at 7.8 mM. After 4–5 days, cells reached ~70% confluence and had a typical epithelial cell morphology. Cells were maintained at 37°C in a humidified incubator with 5% CO_2_/room air, and the medium was changed every 2–3 days up to the time of experimentation, which was typically after 7 days in culture.

### ACE2 Activity Assay

Angiotensin-converting enzyme 2 activity in cell culture media was measured using a commercially available synthetic fluorogenic substrate for ACE2 [Mca-Ala-Pro-Lys(Dnp)-OH] (AnaSpec, San Jose, CA, USA), as we have described (Xiao et al., 2012, 2014). After 48–72 h in culture, cell media was collected, followed by addition of a protease inhibitor cocktail (1% vol/vol) (Sigma, P8340, St. Louis, MO, USA). For all experiments, media was first centrifuged at 10,000*g* for 5 min at 4°C to remove dead cells and cellular debris. Cell media (15 μL) was then added to the wells of a 96-well plate (total volume 100 μL/well) in a solution containing 37.5 mM 2-(*N*-morpholino)ethanesulfonic acid, 225 mM NaCl, 7.5 μM ZnCl_2_, 0.75 mM *N*-ethylmaleimide (NEM), 0.75 mM phenylmethylsulfonyl fluoride (PMSF), 11.25 μM ACE2 substrate, with or without 1 μM of the ACE2 inhibitor MLN-4760 (GL1001, provided by Ore Pharmaceuticals, Cambridge, MA, USA). Samples were protected from light and incubated for 16 h on a plate shaker at room temperature and fluorescence was measured using the FLUOstar Galaxy fluorometer (BMG Labtech., Durham, NC, USA) with excitation wavelength of 320 nM and emission wavelength of 405 nM. ACE2 activity was determined by subtracting the Relative Fluorescence Unit (RFU) obtained in the presence of MLN-4760 from the reading in the absence of inhibitor. ACE2 activity in the culture medium was corrected for the cell protein amounts on the culture dishes, and is reported as RFUs per μg protein per hour (RFU/μg/hr).

### ACE2 Immunoblot Assays

After centrifugation to remove insoluble debris and dead cells, cell culture medium was concentrated with the Amicon Ultra-0.5 30K Centrifugal Filter Devices (EMD Millipore, Darmstadt, Germany). The concentrate (25 μl) was then prepared in a buffer consisting of 31.3 mM Tris-HCl (pH 6.8), 1% wt/vol SDS, 5% glycerol, and 0.025% wt/vol bromophenol blue, and boiled for 5 min. Lysates from proximal tubular cells were prepared in a buffer consisting of 62.5 mM Tris⋅HCl (pH 6.8), 2% wt/vol SDS, 10% glycerol, 50 mM DTT, and 0.01% wt/vol bromophenol blue, boiled for 5 min, and centrifuged at 10,000*g* for 5 min at 4°C to remove insoluble debris.

Twenty-five micro liter of concentrated media (20-fold concentrate) was run on 7.5% SDS-polyacrylamide gels, and subjected to immunoblot analysis using commercially available goat anti-human ACE2 antibodies (1:500 dilution) (AF933, R&D Systems Inc., Minneapolis, MN, USA) as we previously described to characterize mouse shed ACE2 fragments by mass spectrometry (Xiao et al., 2014). Mouse kidney cortex lysates were used as controls (1.5–10 μg protein). Densitometric analysis of the protein bands was performed using Kodak ID image analysis software (Eastman Kodak, Rochester, NY, USA).

### RNA Silencing

Transient transfection of proximal tubular cells was performed with siGENOME SMARTpool silencing (si)RNAs (Dharmacon, Thermo Fisher Scientific, Waltham, MA, USA) using Lipofectamine^TM^ RNAiMAX Transfection Reagent (Invitrogen, Carlsbad, CA, USA) as per the manufacturer’s instructions. Briefly, 60 or 200 pmol scrambled siRNA (Silencer Select negative control #1), PKC-δ siRNA, or PKC-ε siRNA was added to 250 μl Opti-MEM^®^I Reduced Serum Medium (Invitrogen), then added to Lipofectamine^TM^ RNAiMAX that was diluted in 250 μl Opti-MEM, and incubated for 10–20 min at room temperature. The siRNA-Lipofectamine^TM^ RNAiMAX complexes were then added to 35-mm culture dishes containing primary cultures of mouse proximal tubular cells, achieving final siRNA concentrations of 30 nM or 100 nM. ACE2 activity was assayed in the cell culture medium, and PKC-δ or PKC-ε protein expression in cell lysates was assayed by immunoblot 48 h post-transfection.

### Materials

D-glucose and L-glucose were obtained from Sigma. The ADAM17 inhibitor, TNF-α Protease Inhibitor-1 (TAPI-1) was from Calbiochem (San Diego, CA, USA). Go6976 (PKC-α and -β1 inhibitor) and matrix metalloproteinase (MMP)–2, –8, and–9 inhibitors were from EMD Millipore. Ruboxistaurin (PKC-β1 and -β2 inhibitor) and GI250423X (ADAM10 inhibitor) were from Tocris Bioscience (Ellisville, MO, USA). Sotrastaurin (pan-PKC inhibitor) was from Axon Medchem BV (Gronigen, Netherlands). Rottlerin (PKC-δ inhibitor) was from Santa Cruz Biotechnology Inc. (Dallas, TX, USA). Phorbol 12-myristate 13-acetate (PMA) was from Sigma. Antibodies to PKC-δ and -ε were from Cell Signaling (Danvers, MA, USA). RNA silencing nucleotides were from Thermo Fisher Scientific (Waltham, MA, USA). All vehicle controls with use of inhibitors consisted of cells exposed to an equivalent amount of DMSO (0.05%), which in preliminary experiments did not affect ACE2 activity in the media compared to non-DMSO treated cells.

### Statistics

Data are presented as mean ± SE. Data were analyzed using SigmaStat (version 3.5; Systat Software, Inc., San Jose, CA, USA). For multiple comparisons, analysis was by one-way repeated analysis of variance followed by Bonferroni correction. For comparisons involving two groups, Student’s t-test was used. A *p* < 0.05 was considered significant.

## Results

### Effect of D-glucose on ACE2 Shedding in Mouse Proximal Tubular Cells

**FIGURE 1:**
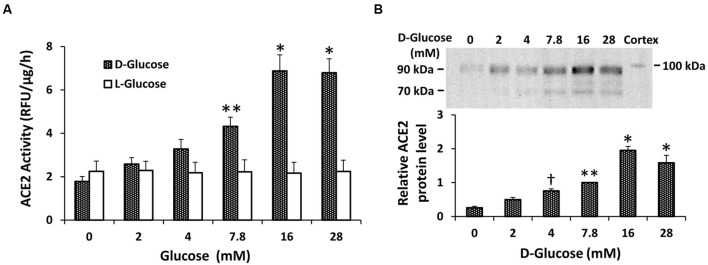
**Concentration-dependent stimulation of ACE2 shedding by D-glucose in mouse proximal tubular cells. (A)** Graph depicts effect of varying concentrations of D- or L-glucose on ACE2 activity recovered from cell culture media. ^∗^*P* < 0.01 vs. 0, 2, 4, and 7.8 mM D-glucose. ^∗∗^*P* < 0.01 vs. 0 mM D-glucose; *n* = 4–5. **(B)** Graph depicts effect of varying concentrations of D-glucose on ACE2 shedding into culture media, determined by immunoblot analysis. Representative immunoblot is depicted above graph, showing ACE2 fragments at 90 and 70 kDa, with mouse kidney cortex ACE2 at 100 kDa. ^∗^*P* < 0.01 vs. 0, 2, 4, and 7.8 mM; ^∗∗^*P* < 0.05 vs. 0 and 2 mM; ^†^*P* < 0.05 vs. 0 mM; *n* = 5.

Initial experiments determined the concentration-dependent effect of D-glucose on ACE2 shedding in mouse proximal tubular cells. As shown in **Figure 1A**, after 72 h ACE2 activity in the media rose progressively with increasing concentrations of D-glucose in the media. This effect was significant at the basal level of 7.8 mM D-glucose (compared to 0 mM D-glucose), and peaked at 16 mM D-glucose. In contrast, increasing concentrations of L-glucose had no effect on ACE2 activity in the media. In separate experiments, varying concentrations of D-glucose in the media alone (in the absence of cells) had no effect on ACE2 activity (*n* = 6, not shown). By immunoblot, shed fragments of ACE2 at 90 and 70 kDa were detected in the media from proximal tubular cells, and these fragments each increased with rising D-glucose concentration (**Figure 1B**).

### Effect of PKC Inhibition on Constitutive ACE2 Shedding

**FIGURE 2:**
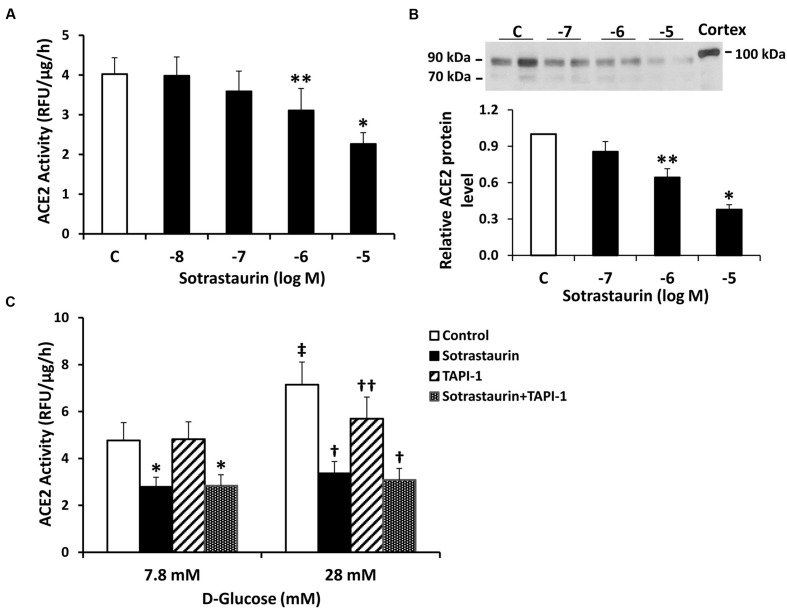
**The pan-PKC inhibitor sotrastaurin reduces ACE2 shedding in mouse proximal tubular cells. (A)** Graph depicts effect of sotrastaurin (10-5-10-8 M) on ACE2 activity recovered from cell culture media. ^∗^*P* < 0.005 vs. all other groups (C, vehicle-treated control); ^∗∗^*P* < 0.05 vs. C and 10^-8^ M; *n* = 6. **(B)** Graph depicts effect of varying concentrations of sotrastaurin on ACE2 shedding into culture media, determined by immunoblot analysis. Representative immunoblot is depicted above graph, showing ACE2 fragments at 90 and 70 kDa, with mouse kidney cortex ACE2 at 100 kDa. ^∗^*P* < 0.01 vs. all other groups; ^∗∗^*P* < 0.05 vs. 10^-7^ M and vehicle-treated control (C); *n* = 5. **(C)** Graph depicts effect of sotrastaurin (10^-5^ M), with or without the ADAM17 inhibitor TAPI-1 (10^-5^ M), on ACE2 activity in the culture media, in basal conditions (D-glucose 7.8 mM) or high D-glucose (28 mM). ^∗^*P* < 0.01 vs. Control and TAPI-1 (7.8 mM D-glucose); In D-glucose 28 mM, ^†^*P* < 0.001 vs. Control and TAPI-1, ^††^*P* < 0.05 vs. Control; ^‡^*P* < 0.001 vs. Control (D-glucose 7.8 mM); *n* = 4–5.

We studied the role of PKC in regulating ACE2 shedding in proximal tubular cells incubated under basal D-glucose conditions (7.8 mM). Incubation of cells with the selective pan-PKC inhibitor sotrastaurin caused a dose-dependent reduction in ACE2 activity and protein in media from cells in 7.8 mM D-glucose (**Figures 2A,B**). While the ADAM17 inhibitor TAPI-1 (10^-5^ M) had no effect on constitutive shedding of ACE2 in basal conditions (7.8 mM D-glucose), it partially blocked stimulation of shedding induced by high glucose, consistent with our previous studies (Xiao et al., 2014) (**Figure 2C**). On the other hand, sotrastaurin (10^-5^ M) completely inhibited ACE2 shedding under conditions of high D-glucose (28 mM), to levels observed under basal conditions, with or without TAPI-1 (**Figure 2C**). In mouse proximal tubular cells maintained in 7.8 mM D-glucose, administration of the activator of PKC, PMA (10^-7^ or 10^-6^ M) for 30 min increased ACE2 activity in the cell culture media by 192 and 209%, respectively (*n* = 2 for each).

**FIGURE 3:**
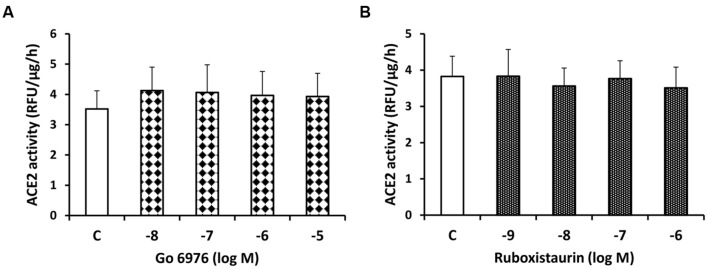
**Lack of inhibitory effect of the PKC-α and -β1 inhibitor Go6976 (A) and the PKC-β1 and β-2 inhibitor ruboxistaurin (B) on constitutive ACE2 shedding in mouse proximal tubular cells.** Graphs depict dose-dependent effects of Go6976 and ruboxistaurin on ACE2 activity in the culture media, compared to vehicle-treated cells (C); *n* = 4 experiments each for **(A,B)**.

**FIGURE 4:**
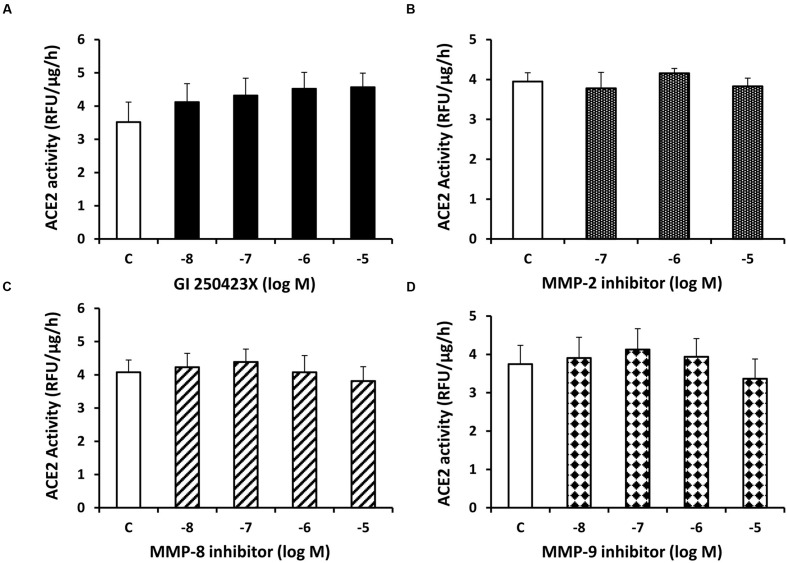
**Inhibition of ADAM10 or matrix metalloproteinases (MMPs)-2, -8, and-9 has no effect on constitutive ACE2 shedding in mouse proximal tubular cells.** Graphs depict dose-dependent effects of the ADAM10 inhibitor GI250423X **(A)**, MMP-2 inhibitor **(B)**, MMP-8 inhibitor **(C)**, and MMP-9 inhibitor **(D)** on ACE2 activity in the culture media, compared to vehicle-treated cells (C); *n* = 4–5 experiments each for **(A–D)**.

In contrast to sotrastaurin, neither the PKC-α and -β1 inhibitor Go6976, nor the PKC-β1 and -β2 inhibitor ruboxistaurin had any effect on ACE2 shedding in cells incubated in 7.8 mM D-glucose (**Figure 3**). Similarly, the ADAM10 inhibitor GI250423X, or inhibitors of MMP-2, -8, or -9 did not affect ACE2 shedding from proximal tubular cells in 7.8 mM D-glucose (**Figure 4**).

### Role of PKC-δ in Constitutive ACE2 Shedding

**FIGURE 5:**
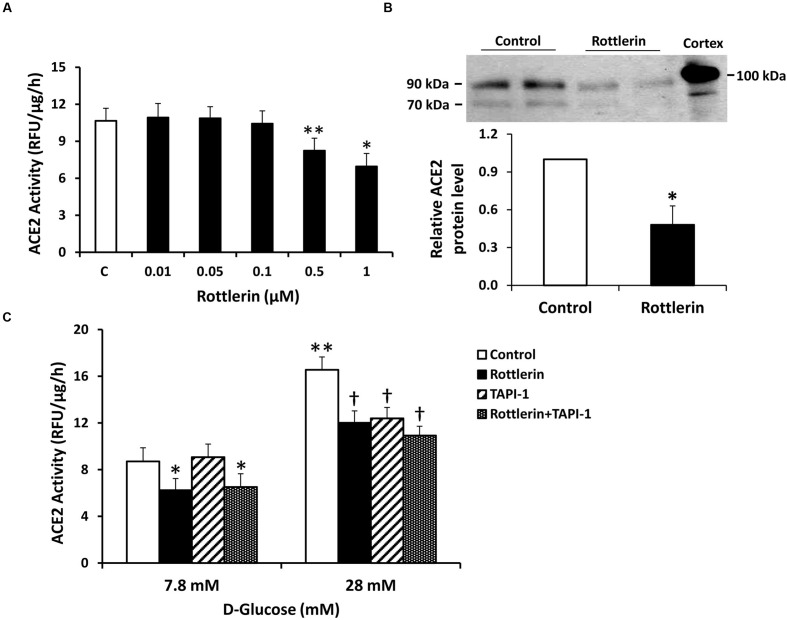
**The PKC-δ inhibitor rottlerin reduces constitutive and high D-glucose-induced ACE2 shedding in mouse proximal tubular cells. (A)** Graph depicts effect of rottlerin (0.01–1 μM) on ACE2 activity recovered from cell culture media, in D-glucose 7.8 mM. ^∗^*P* < 0.001 vs. all other groups (C, vehicle-treated control); ^∗∗^*P* < 0.005 vs. C, 0.01 μM, 0.05 μM, and 0.1 μM; *n* = 5–6. **(B)** Graph depicts effect of rottlerin (1 μM) on ACE2 shedding into culture media, determined by immunoblot analysis. Representative immunoblot is depicted above graph, showing ACE2 fragments at 90 and 70 kDa, with mouse kidney cortex ACE2 at 100 kDa. ^∗^*P* < 0.05 vs. vehicle treated control (C); *n* = 4. **(C)** Graph depicts effect of rottlerin (1 μM), with or without the ADAM17 inhibitor TAPI-1 (10^-5^ M), on ACE2 activity in the culture media, in basal conditions (D-glucose 7.8 mM) or high D-glucose (28 mM); ^∗^*P* < 0.001 vs. Control and TAPI-1, in D-glucose 7.8 mM; ^†^*P* < 0.001 vs. Control, in D-glucose 28 mM; ^∗∗^*P* < 0.001 vs. Control (7.8 mM D-glucose); *n* = 9.

To further probe the role of PKC isoforms in stimulating ACE2 shedding under normal glucose conditions, proximal tubular cells were incubated with the selective PKC-δ inhibitor rottlerin. As shown in **Figure 5**, rottlerin significantly inhibited shedding of ACE2 fragments, as determined by enzyme activity assay and immunoblots. Rottlerin also completely blocked stimulation of ACE2 shedding in high glucose (28 mM). In separate experiments, incubation of cells in high glucose induced no significant difference in total cell lysate expression of PKC-δ, compared to normal glucose (not shown, *n* = 6). The ADAM17 antagonist TAPI-1 had no effect on ACE2 shedding in 7.8 mM D-glucose, but inhibited high glucose-mediated ACE2 shedding (**Figure 5C**).

**FIGURE 6:**
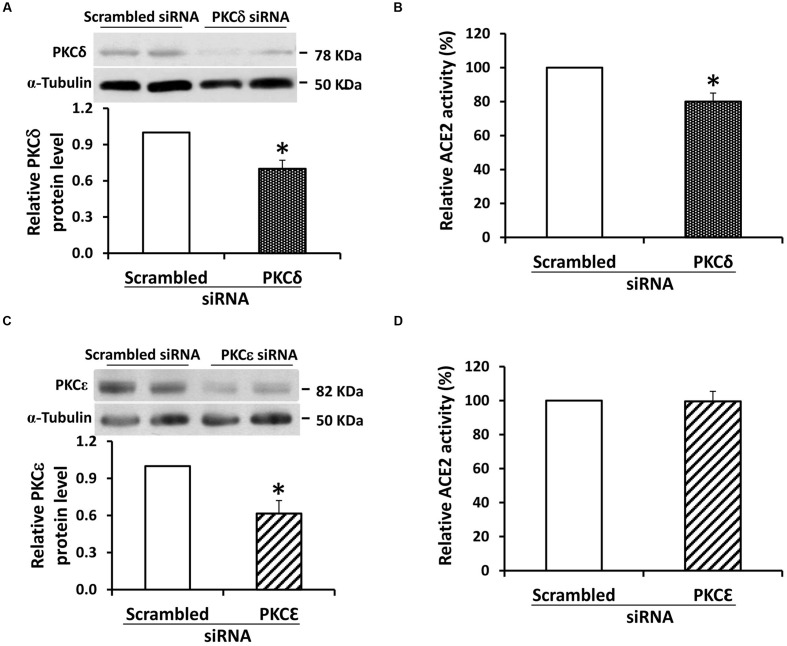
**siRNA knockdown of PKC-δ inhibits constitutive ACE2 shedding in mouse proximal tubular cells. (A)** Graph depicts effect of transfection of siRNA against PKC-δ (100 nM) on protein expression of PKC-δ in mouse proximal tubular cells. Results are depicted as PKC-δ protein relative to cells transfected with scrambled siRNA sequence. Representative immunoblot is shown above graph, with PKC-δ at 78 kDa, and α-tubulin loading control at 50 kDa. ^∗^*P* < 0.05; *n* = 4. **(B)** Graph depicts effect of transfection of siRNA against PKC-δ (100 nM) on ACE2 activity in cell culture media. Results are depicted as ACE2 activity relative to cells transfected with a scrambled siRNA sequence. ^∗^*P* < 0.05; *n* = 4. **(C)** Graph depicts effect of transfection of siRNA against PKC-ε (30 nM) on protein expression of PKC-ε in mouse proximal tubular cells. Results are depicted as PKC-ε protein relative to cells transfected with scrambled siRNA sequence. Representative immunoblot is shown above graph, with PKC-ε at 82 kDa, and α-tubulin loading control at 50 kDa. ^∗^*P* < 0.02; *n* = 6. **(D)** Graph depicts effect of transfection of siRNA against PKC-ε (30 nM) on ACE2 activity in cell culture media. Results are depicted as ACE2 activity relative to cells transfected with a scrambled siRNA sequence; *n* = 7.

RNA silencing was used to further study the role of PKC-δ in ACE2 shedding. In mouse proximal tubular cells grown in 7.8 mM D-glucose, silencing of mRNA for PKC-δ was associated with a modest reduction in protein expression of PKC-δ, yet this caused a significant (20%) decrease in ACE2 activity in the culture media (**Figures 6A,B**). By contrast, while silencing of RNA for the PKC-ε isoform caused a significant 38.5% reduction in protein expression in proximal tubular cells, this had no effect on ACE2 activity in the culture media (**Figures 6C,D**).

## Discussion

In this study we investigated the mechanisms for constitutive proteolytic shedding of ACE2 fragments from mouse proximal tubular cells. Exposure of cells to increasing concentrations of D-glucose caused dose-dependent stimulation of shedding of ACE2 fragments of 90 and 70 kDa. Consistent with our previous report (Xiao et al., 2014), inhibition of ADAM17 had no effect on basal ACE2 shedding (7.8 mM D-glucose), but inhibited high glucose-stimulated shedding (28 mM D-glucose). Here, using pharmacologic blockade and RNA silencing, we show that inhibition of PKC-δ significantly blocks both basal- and high D-glucose-stimulated shedding. The results suggest that PKC-δ is an upstream regulator of ACE2 shedding in proximal tubular cells, and is linked to downstream activation of ADAM17 in high glucose.

Experimental and human diabetes are associated with high urinary levels of soluble active ACE2 fragments, implicating a role for ACE2 shedding as a biomarker of disease activity (Mizuiri et al., 2011; Xiao et al., 2012; Chodavarapu et al., 2013; Park et al., 2013; Wysocki et al., 2013; Cherney et al., 2014; Salem et al., 2014). Indeed, in young adults with type 1 diabetes, urinary ACE2 levels are significantly elevated before development of albuminuria or other clinical complications, compared to healthy control subjects (Cherney et al., 2014). The presence of increased urinary ACE2 fragments in the absence of albuminuria, coupled with evidence that infusion of recombinant ACE2 in mice with diabetes does not increase urinary ACE2 levels (Wysocki et al., 2013), suggests that ACE2 shedding from the apical surface of tubular epithelial cells accounts for a major component of urinary ACE2 content. Significantly increased urinary ACE2 levels were reported in insulin-resistant subjects with impaired fasting glucose, glucose intolerance, or type 2 diabetes, compared to subjects with normal glucose tolerance, suggesting a close link between ACE2 shedding and glucose control (Park et al., 2013). The mechanism for enhanced urinary ACE2 shedding in diabetes appears to involve activation of the sheddase ADAM17. In cultured proximal tubular cells, ADAM17 activity is increased by exposure to high glucose (Xiao et al., 2014). Renal expression of ADAM17 is enhanced in experimental models of type 1 and 2 diabetes, and restoration of normoglycemia inhibits renal ADAM17 expression and reduces urinary ACE2 shedding (Chodavarapu et al., 2013; Salem et al., 2014). Consistent with these studies, our results indicate that exposure to high D-glucose stimulates ACE2 shedding from proximal tubular cells, an effect blocked by TAPI-1, an inhibitor of ADAM17. The data further suggest the existence of a glucose concentration threshold for activation of ADAM17 and stimulation of ACE2 shedding, since blockade of ADAM17 had no impact on ACE2 shedding under basal glucose conditions.

Proteolytic shedding of cell surface proteins is a well-described phenomenon involved in regulation of protein expression and may result in release of enzymatically active peptide fragments. Ectodomain cleavage of ACE2 was first described in ACE2-transfected HEK293 cells and Huh7 cells, and shedding was stimulated by phorbol esters, an effect blocked by ADAM17 inhibition (Lambert et al., 2005). In agreement with our data, ADAM17 inhibition had no effect on basal ACE2 shedding in that study. However, in cultured human airway epithelial cells, constitutive shedding of soluble ACE2 fragments was significantly decreased by inhibition of ADAM17 (Jia et al., 2009) and in COS7 cells stably expressing silencing RNA constructs against endogenous ADAM17, ACE2 shedding was reduced (Grobe et al., 2015). Gene deletion of ADAM17 in Chinese Hamster Ovary (CHO) cells and fibroblasts was associated with reduced constitutive shedding of the larger of the two soluble ACE2 fragments, an effect that was rescued by reconstitution of ADAM17 in these cells (Iwata et al., 2009). These studies highlight the complexity of pathways involved in proteolytic cleavage of ACE2, which may be affected by cell-specific regulatory signals, or perhaps ambient glucose concentration.

In our previous studies in mouse proximal tubular cells, we demonstrated the presence of two major shed ACE2 fragments at 90 and 70 kDa, with mass spectrometric identification at amino acid positions 18–706 and 18–577, respectively, (Xiao et al., 2014). The appearance of each ACE2 fragment increased progressively with rising D-glucose concentration in the present studies, and constitutive shedding of both fragments was reduced by a pan-PKC inhibitor, but not by inhibitors of isoforms of PKC-α, -β1, or -β2, ADAM10, or 3 other MMPs. PKC isoforms are classified into three distinct groups: conventional PKC isoforms (PKC-α, -β1, -β2, and -β) are sensitive to diacylglycerol (DAG) and calcium, novel PKC isoforms (PKC-δ, -ε, -𝜃, and -η) are sensitive to DAG but insensitive to calcium, and atypical PKC isoforms (PKC-ζ, and-ι/λ) are not regulated by DAG or calcium (Parker and Murray-Rust, 2004). In the present studies, siRNA against PKC-δ caused a modest 30% reduction in its protein expression, but this induced a significant decrease in constitutive ACE2 shedding, while knockdown of PKC-ε had no effect on shedding. These results support a unique role for PKC-δ in constitutive ACE2 shedding in these cells, and are consistent with involvement of PKC-δ in constitutive or phorbol ester-stimulated shedding of the ectodomain of membrane-bound heparin-binding EGF in other studies (Izumi et al., 1998; Uchiyama et al., 2009; Kveiborg et al., 2011). In human myeloma cells, PKC-δ has also been shown to regulate phorbol ester-induced shedding of the interleukin-6 receptor (Thabard et al., 2001). Although our study did not identify the metalloprotease responsible for PKC-δ-stimulated basal ACE2 shedding in proximal tubular cells, potential candidates include ADAM9 and ADAM12, which have been identified as signaling partners of PKC-δ (Izumi et al., 1998; Murphy, 2009). Moreover, since inhibition of PKC-δ also blocked high glucose-induced ACE2 shedding, it is possible that high glucose-stimulated DAG may be involved in activation of PKC-δ, and downstream activation of ADAM17 in these cells. Although we did not detect changes in total cell PKC-δ expression by immunoblot with high glucose, further studies are necessary to determine if high glucose stimulates translocation of PKC-δ from the cytosolic to the plasma membrane fraction in proximal tubular cells.

The impact of ACE2 shedding from proximal tubular cells on local degradation of Ang II and generation of Ang-(1–7) is poorly understood. In this regard, in mice ADAM17-mediated shedding of ACE2 from brain neurons contributes to the development of neurogenic hypertension (Xia et al., 2013), suggesting that preservation of membrane-bound ACE2 may be important in counteracting local activation of the RAS. Thus, targeting PKC-δ in proximal tubular cells could represent a strategy to prevent urinary ACE2 shedding under basal or high glucose conditions, thereby maintaining membrane-bound ACE2 as a brake on the local RAS. Studies in mouse models of diabetes or proteinuria have shown that genetic deletion of PKC-δ protects renal tubular cells against apoptosis (Li et al., 2010), and in mice treated with the nephrotoxin cisplatin, gene deletion or pharmacologic blockade of PKC-δ reduces kidney injury and tubular cell apoptosis (Pabla et al., 2011). Accordingly, the potential role of reduced shedding of ACE2 from proximal tubular cells in these protective responses merits further study.

In summary, our results indicate that inhibition of PKC-δ in mouse proximal tubular cells reduces constitutive proteolytic shedding of ACE2, and blocks high glucose- or ADAM17-stimulated ACE2 shedding. The data suggest that PKC-δ is a critical upstream regulator of ACE2 shedding in these cells. Further *in vivo* animal studies are required to test the role of PKC-δ in mediating ACE2 shedding from proximal tubule and to confirm the *in vitro* findings presented here.

## Author Contributions

FX and JZ performed all the experimental work. FX, JZ, and KB designed the studies and conducted all statistical analyses. All authors were involved in the interpretation of the data. KB wrote the first draft of the manuscript and all authors contributed to critical revisions. All authors contributed and approved the final manuscript.

## Conflict of Interest Statement

The authors declare that the research was conducted in the absence of any commercial or financial relationships that could be construed as a potential conflict of interest.
